# A hybrid ResNet50-vision transformer model with an attention mechanism for aerial image classification

**DOI:** 10.1038/s41598-026-36492-4

**Published:** 2026-02-11

**Authors:** Amr Aboghanem, Mohamed Abd Elfattah, Hanan M. Amer, Abeer Tawkol Khalil

**Affiliations:** 1 Electronics and Communications Department, Faculty of Engineering, Mansoura University, Mansoura University, Mansoura, 35516 Egypt; 2 Computer Science Department, Misr Higher Institute for Commerce and Computers, Mansoura, 35511 Egypt

**Keywords:** Aerial images, Image classification, Vision transformer (ViT), Attention mechanism, Climate sciences, Natural hazards, Health care, Risk factors, Energy science and technology, Engineering

## Abstract

Aerial image classification is considered an open challenge due to its properties and the presence of various complex images. Given the complexity and variation in aerial images, this paper proposes two hybrid models for classification. The first hybrid model combines features extracted from ResNet-50 and the Vision Transformer (ViT), followed by the application of multi-head attention (MHA) to detect the most informative features. The second hybrid model also extracts features from ResNet-50 and ViT, then applies cross-attention. Both hybrid models are assessed using the benchmark Sikkim Aerial Images Dataset for Object Detection (SAIOD). The efficacy of the two hybrid models is assessed using the well-established performance metrics, including precision, recall, F1-score, and the ROC curve. The results indicate that the first model, which employs MHA, achieves superior performance with an accuracy of 95.80%. Both models outperform the best existing methods, achieving accuracies of 95.80% and 95.52%, respectively.

## Introduction

The classification of aerial images has lately attracted significant attention due to the wide variety of applications it has. Some of these applications include the identification of natural hazards, urban planning, disaster management, and environmental monitoring.^1^. The growth in aerial imaging market size is expected to increase from USD 3.39 billion in 2025 to USD 7.43 billion by 2030, with the CAGR at 16.98%, according to^2^. As stated by the Union of Concerned Scientists (UCS)^3^, there are 7,560 satellites currently orbiting Earth, involving their nation of origin, principle, and additional functioning details.

The tremendous and unprecedented development of deep learning model applications, along with the machine’s learning-based methods, achieved outstanding performance in solving many challenges, including computer vision and its applications, especially dealing with the classification and identification of aerial images with high efficiency, and contributed accurately to addressing many issues sustainably. Examples of these applications are agricultural and industrial applications, target tracking in military applications, and real-time response capabilities. Additionally, it can be used to define geopolitical boundaries and sustainably benefit maritime applications. It is worth noting that assistance can be provided in real-time transaction-based applications in smart cities in a sustainable manner to achieve the Sustainable Development Goals.

There are many state-of-the-art methods that have been used to deal with the various processes of these aerial images, an innovative convolutional neural network (CNN) architecture for the recognition of cacti from aerial images was proposed^4^. The cloud-based pipeline improves training efficiency by providing scalable data storage, preprocessing, and distributed training across platforms such as Amazon Web Services (AWS), Google Cloud Platform (GCP), and Microsoft Azure. The model incorporates residual connections and depthwise separable convolutions, attaining 96.7% accuracy on the aerial cactus recognition dataset. The method’s superior performance, cost-effectiveness, and scalability make it appropriate for practical aerial picture categorization applications.

A review article examines YOLO, Faster R-CNN, U-Net, and ResNet, four deep learning approaches that are often used to recognize animals in satellite and aerial images was introduced^5^. Challenges include unbalanced datasets, limited samples, image annotation, and uncertainty estimates. The proposed solutions include sample annotation techniques, self-supervised learning, and network architectures. Emerging research themes include video-based detection, high-resolution satellite imagery analysis, multispecies detection, and innovative annotation techniques^5^.

The emergence of modern technology has considerably enhanced both the amount and quality of aerial imagery. As a result, deep neural networks (DNNs) have emerged as the predominant method for aerial scene categorization tasks owing to their exceptional capacity to extract high-quality feature representations^1^. The advancement of deep learning-based models plays a vital role in recent applications^6^, especially in the image classification field, where all the models that were built on this technology achieved remarkable results. Deep learning models have markedly advanced over the last decade, driven by their rising use in diverse fields and the increasing recognition of their social consequences. During the early stages of deep learning research, the emphasis was mostly on achieving high predicted accuracy and model complexity, sometimes overlooking the possible biases included in the data and techniques used. Nonetheless, as the use of deep learning applications expanded, researchers and practitioners emerged^7^.

A detailed evaluation of recent developments in lightweight deep learning models designed for aerial scene categorization was introduced in^1^. The authors categorize these models based on the methods they employed to reduce their weight. An in-depth examination of each category’s performance on benchmark datasets is offered here, along with a discussion of the benefits and drawbacks associated with each category. These include the prospects for future research in this field and the challenges researchers face^1^.

To compare learning at the object and image levels, a slot attention-based Generalized Category Discovery (GCD) training procedure (Slot-GCD) was proposed in^8^. Extensive tests on three publicly available aerial picture datasets demonstrate the method’s superiority over the most advanced techniques. Specifically, using the Aerial Image data set (AID) dataset, Slot-GCD attains 91.5% recognition accuracy for known old classes and 81.9% for unknown novel class data. In^9^, a lightweight and explainable CNN was designed for emergency monitoring from aerial imagery. It achieved classification of 96% accuracy on the Aerial Image Database for Emergency Response (AIDER). Mogaka et al.^10^ introduced an optimized EmergencyNet CNN model on resource-constrained UAVs. Additive powers-of-two (APoT) quantization was applied to further reduce the model size and improve computational efficiency. It was evaluated on the Aerial Image Disaster Event Recognition (AIDER) dataset. It achieved an F1-score of 93.6% with 4-bit APoT quantization, which closely matches approaches the full precision (32-bit) accuracy of 94%.

In^11^, a comparison between U-Net, Fully Convolutional Network (FCN), DeepLabv3+, and SegNet was conducted on the data from the North Rhine-Westphalia geoportal; the numerical results affirm that the CNN was better at discriminating between solid (asphalt, concrete) and modular (paving stones, tiles) surfaces for both sidewalks and roads on aerial data of spatial resolution of 10 cm. U-Net is the best model with an accuracy of 92%.

A novel model based on an optimized hierarchical vision transformer was proposed for aerial image classification named SwinSight^12^, in which the computational challenges were addressed based on the shifted window mechanism and applied to the SAIOD dataset with 93.16% accuracy.

A novel framework was recently proposed in^13^ wherein the YOLO11 was integrated with a custom Feature Enhancement Module (FEM), created to enhance harsh features through the use of universal MLP fusion, multi-head channel attention, and expounded convolutions. The accuracy was 95.33% on the SAIOD dataset.

Many recently published studies rely heavily on CNN and ViT, or a combination of both, due to their strength in extracting discriminative features and the effective role of the attention mechanisms for developing new effective models across various fields^14–18^.

Aerial images are not the same as images of natural scenes. Usually, the targets that need to be found are small, vary significantly in size, are obscured, have no clear direction, are densely distributed. These difficulties arise when attempting to identify targets in aerial images^19^. Therefore, we are motivated to contribute to the development of this field, which has attracted significant attention through classification models. To the best of our knowledge, this study is the first to apply a hybrid deep learning model to the SAIOD dataset, which integrates the ResNet-50 and Vision Transformer (ViT) features with attention mechanisms. The paper’s contributions could be summarized as follows:

- This work presents a combination of the well-known pre-trained model ResNet-50 with the ViT Transformer, which significantly contributes to achieving high accuracy for aerial image classification. This merging supports vast potential for dangerous applications like disaster response and reformatting the future of computer vision technologies and will be advantageous for applications in various areas.
- In this work, deep learning models are designed and carefully analyzed to differentiate between ten distinct classes, which is a challenging task that is believed to be complicated.
- Two hybrid models are proposed based on the power of multi-head attention (MHA) and cross-attention. which are employed for detecting the most significant features then followed by the fully connected layers for the classification phase, which help to achieve the highest classification accuracy.
- A set of experiments was used to evaluate the proposed model. Well-known metrics were used, such as F1-score, precision, recall, accuracy, and the ROC curve. The numerical results indicate that the proposed model is better at classifying aerial images, which will be useful in the real world for things like environmental monitoring, object detection, and military use, etc...

This article is structured as follows: Sect. 2 presents the preliminaries. The proposed model is illustrated and discussed in detail in Sect. 3. The results of the experiments are presented and analyzed in Sect. 4. The paper is concluded, and future work is presented in Sect. 5.

## Preliminaries

### Residual network (ResNet-50)

ResNet-50 was first presented in^20^, and the residual building block (RBB) mentioned in^21^ is a key feature that allows some neural layers to be bypassed using shortcut connections. This makes the best use of trainable parameters in error backpropagation, prevents slopes from vanishing or growing, and builds a deeper CNN structure, which eventually makes classification work better. The structure (ResNet-50) includes 49 Conv layers, a fully connected layer (FC), and a softmax classifier. It has a depth of 51 layers and is designed to improve its classification prediction accuracy. The depth of the network layers and better feature extraction layers are expected to enhance the final prediction accuracy. The ResNet-50 is employed as a pre-trained model with the same pre-trained weights. The main objective of the ResNet-50 in our hybrid model is to extract the most important features.

RBB is composed of several batch normalizations (BN), ReLU activation functions, convolutional layers (Conv), and one shortcut. There are two different blocks of RBB structures^21^. In RBB-1, let *M* stand for the nonlinear function that controls the neural path. The output of RBB-1 can be expressed as in Eq. (1).

1 $$\begin{aligned} C = M\left( x \right) + x \end{aligned}$$

The output of RBB-2 can be expressed in Eq. (2) by denoting *H* as the shortcut path.

2 $$\begin{aligned} C = M\left( x \right) + H\left( x \right) \end{aligned}$$

### Vision transformer (ViT)

The Vision Transformer (ViT) was introduced by Dosovitskiy et al^22^ in 2020, in which the images are represented as a sequence of tokens, and the standard transformer architecture is used in the processing and classification phases. The transformer divides each image into a grid of non-overlapping patches, which serves as its input. A 1*D* chain of token embeddings is fed into the standard Transformer. To work with 2*D* images like the ones in our article, the image was reshaped into a series of flattened 2D patches $\textbf{x}_p \in \mathbb {R}^{N \times (P^2 \cdot C)}$, where (*H*, *W*) is the original image’s resolution, *C* is the number of channels, (*P*, *P*) is the resolution of each patch, and $N = HW/P^2$ is the number of patches. This is also the length of the input sequence for the Transformer. The Transformer’s latent vector size *D* stays the same across all of its layers, the patches were flattened, and the trainable linear projection was used Eq. (3) for mapping them to *D* dimensions^22^. As mentioned in^22^, the MLP contains two layers with a GELU non-linearity

The Transformer employs a constant latent vector size *D* throughout all of its layers. Consequently, then flatten the regions and map them to *D* dimensions using a trainable linear projection, Eq. (3).

Eq (6) uses a learnable embedding to the sequence of embedded patches $(Z^0_0 = x_{class})$, which is comparable to BERT’s [class] token. The image representation *y* is represented by the state at the transformer encoder’s output $Z^0_L$.

As shown in Eqs. (4, 5), the Transformer encoder^23^ is consists of alternating layers of multiheaded self-attention and MLP blocks, detailed in Ref^22^.

3 $$\begin{aligned} z_0= & [x_{\text {class}}; x_p^1 \textbf{E}; x_p^2 \textbf{E}; \cdots ; x_p^N \textbf{E}] + \textbf{E}_{pos}, \quad \textbf{E} \in \mathbb {R}^{(P^2 \cdot C) \times D}, \, \textbf{E}_{pos} \in \mathbb {R}^{(N+1) \times D} \end{aligned}$$

4 $$\begin{aligned} z'_\ell= & \text {MSA}(\text {LN}(z_{\ell -1})) + z_{\ell -1}, \quad \ell = 1 \ldots L \end{aligned}$$

5 $$\begin{aligned} z_\ell= & \text {MLP}(\text {LN}(z'_\ell )) + z'_\ell , \quad \ell = 1 \ldots L \end{aligned}$$

6 $$\begin{aligned} \textbf{y}= & \text {LN}(z_L^0) \end{aligned}$$

In the ViT environment, each image is split into patches, a process shown in Eq. (7).

7 $$\begin{aligned} \text {Patches} = \{x_1, \ldots , x_N\}, \quad x_i \in \mathbb {R}^{P^2 \cdot C} \end{aligned}$$

Where $P = 16$, and $N = \frac{224^2}{16^2} = 196$. Each patch is embedded and encoded, as shown in Eq. (8).

8 $$\begin{aligned} z_0^i = E \cdot x_i + p_i \end{aligned}$$

The ViT output:

9 $$\begin{aligned} f_{\text {ViT}} = \text {ViT}(I'') \in \mathbb {R}^{196 \times 768} \end{aligned}$$

## Proposed model for aerial image classification

**Fig. 1:**
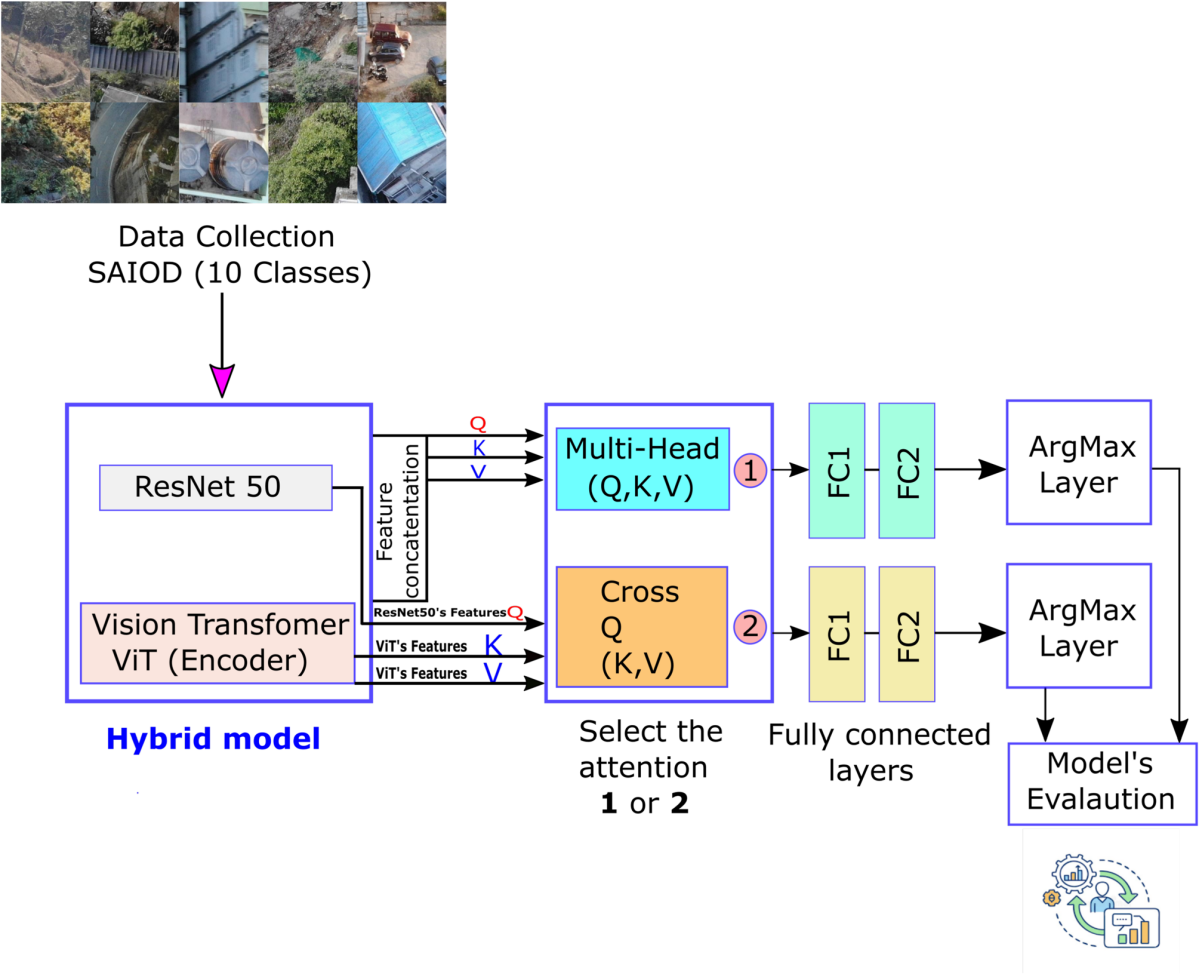
The proposed workflow for the two hybrid models.

This section describes the two suggested hybrid models which are composed of three steps. The first step is to pull out important features from the ResNet-50 and ViT (encoder). The second step is to use MHA or cross-attention to identify the best features. Lastly, the third step uses two fully connected layers and the argmax function to complete the classification task. The proposed workflow is illustrated in Fig. 1.

The two hybrid models are illustrated in Fig. 1, starting with reading the dataset (see Sect. 4.1), which has 10 different classes, each with unique properties, and then examining its features. Each image is represented by $I \in \mathbb {R}^{H \times W \times C}$ and is resized to $I' \in \mathbb {R}^{224 \times 224 \times 3}$, where the variables *H*, *W*, *andC* refer to height, width, and color, respectively. The equation below refers to the output of the ResNet-50.

10 $$\begin{aligned} f_{\textrm{cnn}} = \mathrm {ResNet-50}(I'') \in \mathbb {R}^{2048} \end{aligned}$$

The first hybrid model is based on the feature concatenation from the ResNet-50 and ViT (encoder), followed by the MHA. The *Q*,*K*,*V* served as input to MHA are determined by the concatenation of features, which comes from ResNet-50 and ViT. MHA enables the model to concurrently concentrate on input from various representation subspaces at separate positions^23^. A mapping of the values of *Q*, *K*, and *V* could be the main purpose of an attention function, while the concatenated of these values is considered the output^23^. For MHA, the following Equations below treat it as a single-token sequence:

11 $$\begin{aligned} X = f_{\textrm{combined}} \;\in \; \mathbb {R}^{1 \times 2816} \end{aligned}$$

Here, the $f_{\textrm{combined}}$ refers to the concatenated ResNet-50-ViT vector.

Define:

12 $$\begin{aligned} Q = X W^Q,\quad K = X W^K,\quad V = X W^V \end{aligned}$$

Then:

13 $$\begin{aligned} \textrm{Attention}(Q,K,V) = \textrm{softmax}\left( \frac{QK^\top }{\sqrt{d_k}}\right) V \end{aligned}$$

MHA output:

14 $$\begin{aligned} A = \textrm{MultiHead}(X) \in \mathbb {R}^{1 \times 2816} \end{aligned}$$

After squeezing:

15 $$\begin{aligned} A' = \text {squeeze}(A) \in \mathbb {R}^{2816} \end{aligned}$$

For the second hybrid model, all steps are the same as with the first hybrid model, except for the determination of the *Q*, *K* and *V* values for cross-attention; the following Equations are employed:

for the Cross-Attention, these inputs are *Q*, *K*, and *V*, Treat CNN output of the ResNet-50 as a single query vector shown in the Eq. (16) below.

16 $$\begin{aligned} Q = f_{\text {CNN}} W^Q \in \mathbb {R}^{1 \times d_q} \end{aligned}$$

while the *K*, and *V* values are come from the ViT tokens output. Eq. (17).

17 $$\begin{aligned} K = f_{\text {ViT}} W^K \in \mathbb {R}^{196 \times d_k}, \quad V = f_{\text {ViT}} W^V \in \mathbb {R}^{196 \times d_v} \end{aligned}$$

Attention weight is computed, as shown in Eq. (13).

The output from model 1 to model 2 is followed by the following steps. The two fully connected layers were used, as described in Eq. (18) below.

18 $$\begin{aligned} h_1 = \textrm{ReLU}(W_1 A' + b_1), \quad W_1 \in \mathbb {R}^{512 \times 2816} \end{aligned}$$

Here, the $h_1$ indicates the output of the fully connected layers after the MHA is applied to the ResNet-50-ViT vector.

19 $$\begin{aligned} & h_1^{\textrm{drop}} = \textrm{Dropout}(h_1) \end{aligned}$$

20 $$\begin{aligned} & \hat{y} = W_2 h_1^{\textrm{drop}} + b_2, \quad W_2 \in \mathbb {R}^{10 \times 512} \end{aligned}$$

$W_1$ and $W_2$ refer to the weights of the first and second layers, respectively.

The following function, named Cross-Entropy Loss ($\mathscr {L}_{\textrm{CE}}$), is used to compute and update the weight of the model during the training process.

21 $$\begin{aligned} \mathscr {L}_{\textrm{CE}} = -\log \left( \frac{e^{\hat{y}_y}}{\sum _{j=1}^{10} e^{\hat{y}_j}} \right) \end{aligned}$$

The Adam optimizer is used in our hybrid model.

22 $$\begin{aligned} \theta \leftarrow \theta \ - \eta \cdot \frac{\partial \mathscr {L}}{\partial \theta } \end{aligned}$$

where $\mathscr {L}$ refers to the learning rate and $\theta$ indicates the model parameters. For computing the accuracy of the model ($Acc_m$), the following function is used, shown in Eq. (23).

23 $$\begin{aligned} \mathrm {Acc_m} = \frac{1}{N} \sum _{i=1}^{N} \textbf{1}\left( \arg \max _j \hat{y}^{(i)}_j = y^{(i)}\right) \end{aligned}$$

Then the evaluation models are conducted in the same environment (See Sects. 4.2 and 4.3). This workflow shows the options of selecting the type of attention to complete and determining the meaningful features that could be advantageous towards achieving the highest accuracy values.

## Experiment

### Dataset

The SAIOD dataset^24^ was created by gathering aerial images from the Indian state of Sikkim. This data was collected using a DJI Mavic Air drone. The landscape consists of rivers, forests, gorges, hills, undulating surfaces, and populated areas. Ten object classes were recognized, including houses, vehicles, trash, pathways, metal roads, open fields, shadows, tanks, trees, and roofs^12^. The dataset collection process is outlined below; from 60 to 120 meters in the air, in both bright and dim light, over 300 high-resolution images were gathered, (detailed in^12^ ). The 270 $\times$ 270 sliced images in the dataset have a 50% overlap. The ground truth information for each class was verified by manual selection. The dataset is publicly available at (https://data.mendeley.com/datasets/vwznrr98b9/1).

**Fig. 2:**
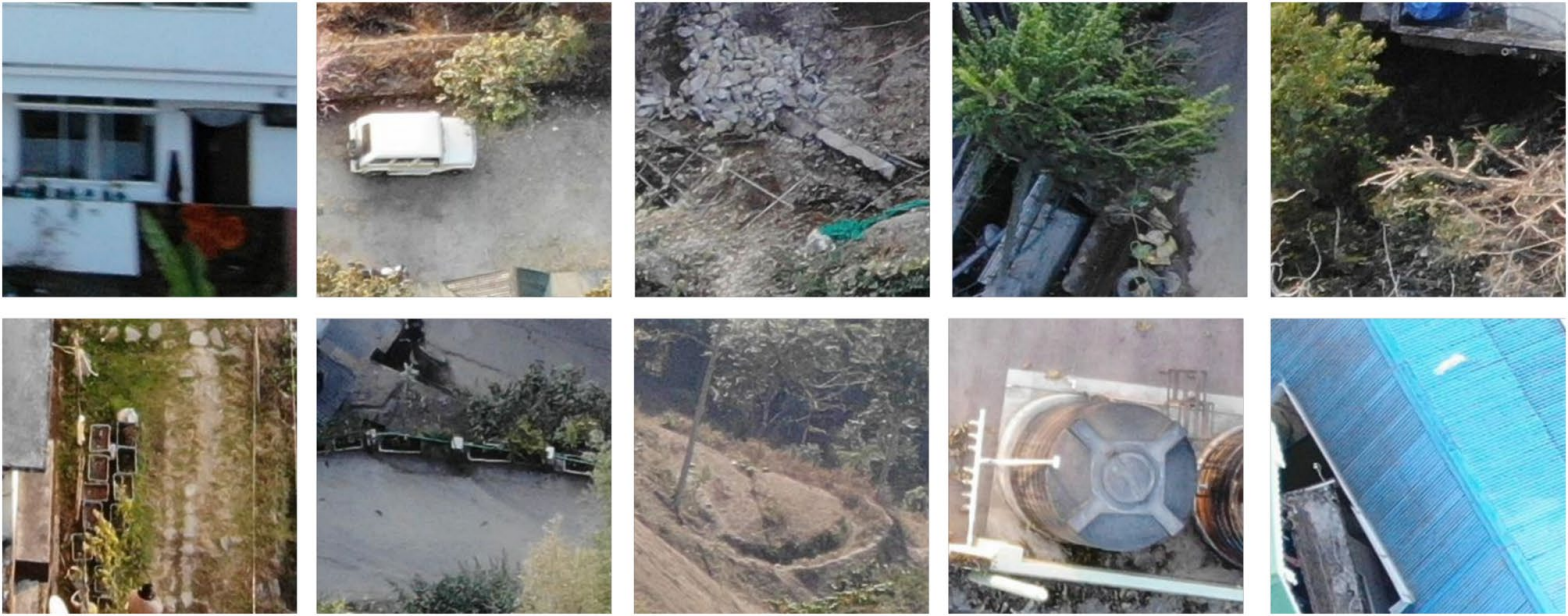
Sample of the SAIOD dataset.

Representative samples were randomly chosen from the SAIOD dataset to visualize the ten different classes. As shown in Fig. 2, each image contains many rich features, and it is considered a multiclass challenge to design an effective model for the classification. Data distribution for the SAIOD dataset is shown in Table 1. The dataset appears balanced, so no oversampling or undersampling techniques were required. Each class contains 1000 images during the training phase, while each class has 250 images in the testing phase.

**Table 1:** Data distribution for the SAIOD dataset.

Class index	Class name	Train set size	Test set size	Total
0	Buildings	1000	250	1250
1	Cars	1000	250	1250
2	Debris	1000	250	1250
3	Footpaths	1000	250	1250
4	Metalled Roads	1000	250	1250
5	Open Fields	1000	250	1250
6	Shadows	1000	250	1250
7	Tanks	1000	250	1250
8	Trees	1000	250	1250
9	Roofs	1000	250	1250
Total		10000	2500	**12500**

Significant values are in bold

### Experiment setup

All experiments are conducted under the same properties; Table 2 summarizes these environmental parameters.

**Table 2:** Two hybrid model’s parameters.

Hardware	
Operating system	Windows 11
System type	64-bit operating system
Platform	Google Colab
Runtime type	Python
Hardware accelerator	T4 GPU
Software	
Optimizer	Adam^25^
No. of Epochs	15
Dropout	0.5
Loss	Categorical$\_$crossentropy
Activation	ReLU
Learning rate	0.0001

### Evaluation protocols

To confirm the efficacy of the proposed model, the well-known performance metrics are used. There are four mathematical equations (Eqs. 24, 25, 26, and 27) that show how to figure out the overall performance of the model. The performance metrics include accuracy, precision, recall, F1 score, confusion matrix, and receiver operating characteristics (ROC)^26^.

- *Accuracy * Accuracy denotes the ratio of samples for which the predicted outcomes align with the actual results over all samples, as follows:
  24 $$\begin{aligned} Accuracy = \frac{TN+TP}{TP+TN+FP+FN} \end{aligned}$$
- *Precision* Precision indicates the proportion of correctly predicted positive instances to the total predicted positive samples.
  25 $$\begin{aligned} Precision= \frac{TP}{FP+TP} \end{aligned}$$
- *Recall* Recall denotes to the ratio of positive class predictions to the total number of positive examples, calculated using the following Eq. (26).
  26 $$\begin{aligned} Recall= \frac{TP}{FN+TP} \end{aligned}$$
- *F1-Score* The F1 score denotes the harmonic average of precision and recall.
  27 $$\begin{aligned} {{F1-Score}}= \frac{2TP}{\left( 2TP+FP+FN \right) } \end{aligned}$$

where the True Positive, True Negative, False Positive, and False Negative are denoted by *TP*, *TN*, *FP*, and *FN*, respectively.

### Numerical results

**Fig. 3:**
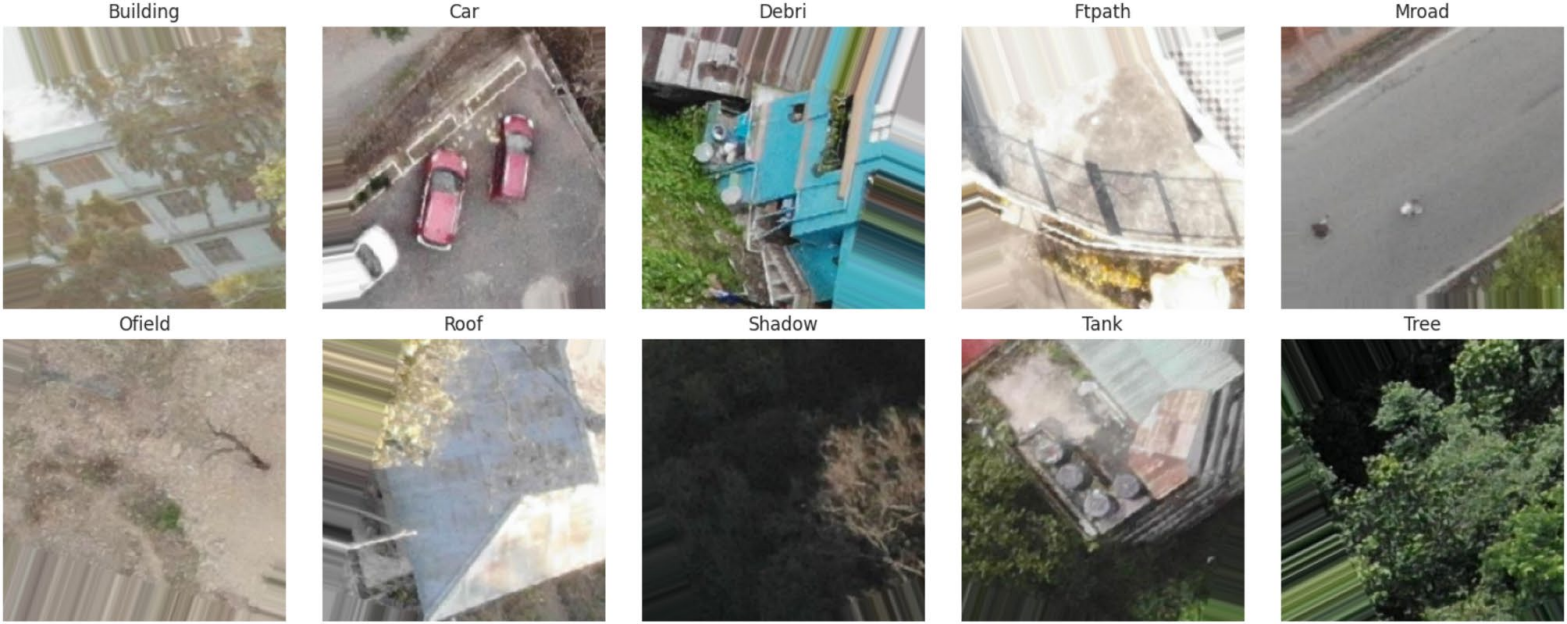
Samples of the SAIOD dataset during the proposed model training.

This section aims to examine how well different types of attention work, as each type can either improve or reduce the hybrid model’s ability to achieve a high classification rate. The main purpose is to select the most accurate or beneficial attention type (like multi-head attention or cross attention). These models are compared under the same environment (See Table 2.). Through the training process, samples of the dataset are picked out randomly and shown in Fig. 3.

### Training/validation (accuracy, loss)

**Fig. 4:**
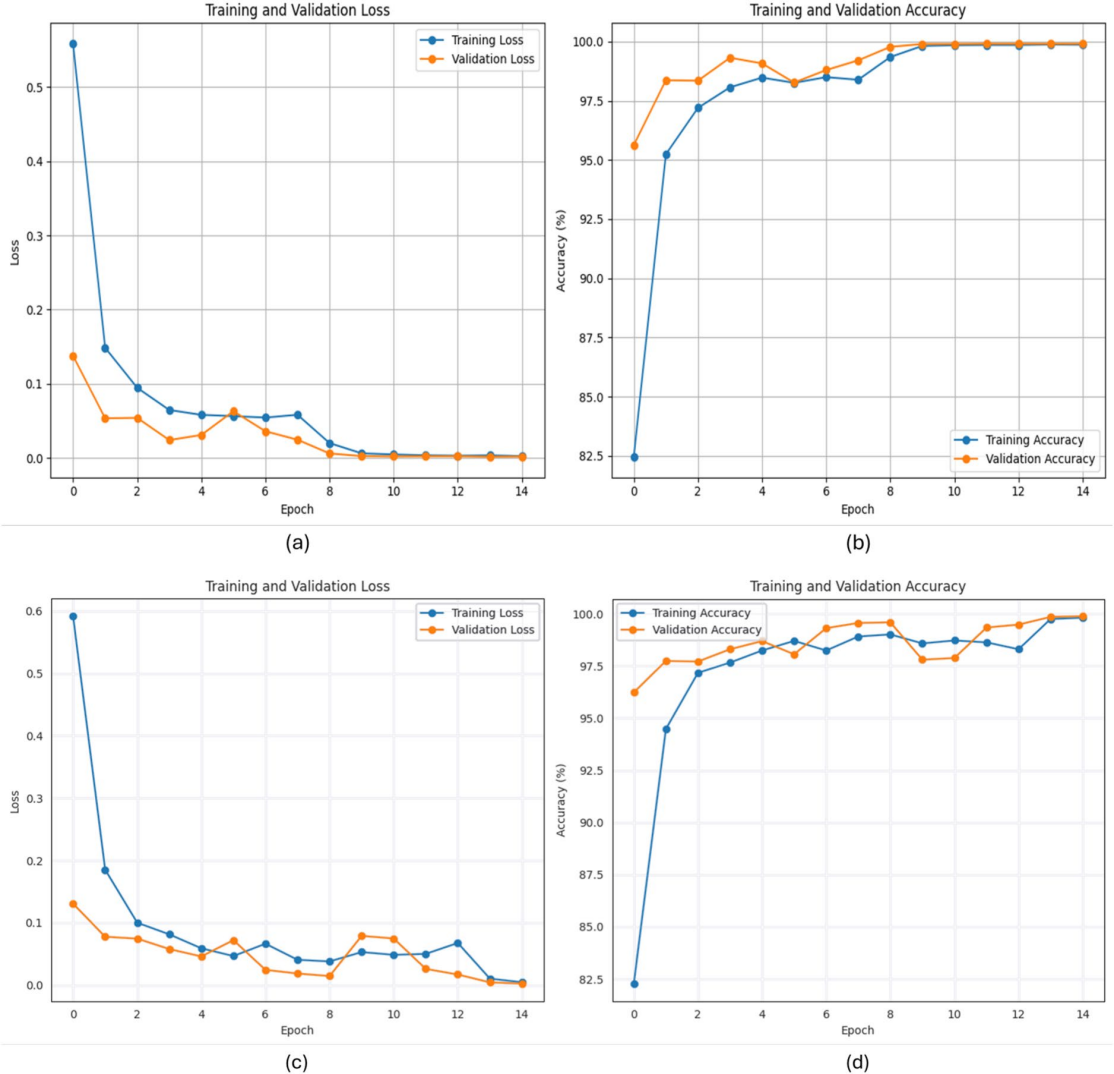
Training/validation (accuracy, loss) of the two hybrid model’s.

Training/validation (accuracy, loss) curves provide an accurate analysis of the behavior of the DL models during the training and validation. Figure 4 illustrates the corresponding curves of the two models. Figure 4a and b illustrate the training loss and accuracy, as well as the validation loss and accuracy for the first hybrid model, respectively. These curves confirm the stability of the proposed model after 10 epochs. In epoch 15, the values are Loss=0.0025, training accuracy=99.87%, validation Loss=0.0019, and validation accuracy=99.91%. Best accuracy so far: 99.91%. Furthermore, for the second model, its curves are shown in Fig. 4 c, d, the Training Loss=0.0047, Training accuracy=99.81%, Validation Loss=0.0025, Validation accuracy = 99.88%, and Best Accuracy So Far: 99.88%. It could be noticed that the first method shows stability after epoch 10, both in accuracy and loss. While the second model exhibits some minor fluctuations towards the end, it is generally stable. Overall, it can be said that the first model is more stable, performs better, and is more sustainable.

**Table 3:** Comparison of the two models in terms of precision, recall, and F1 score values; best values in bold.

Model	Class	Precision	Recall	F1-score
1	Building	0.93	0.96	0.95
2		**0.95**	0.96	0.95
1	Car	0.96	**0.98**	0.97
2		**0.98**	0.96	0.97
1	Debris	**0.96**	**0.94**	**0.95**
2		0.97	0.92	0.94
1	Ftpath	**0.95**	0.93	0.94
2		0.94	**0.95**	0.94
1	Mroad	**0.96**	0.97	**0.97**
2		0.93	**0.98**	0.95
1	Ofield	**0.96**	**0.94**	**0.95**
2		0.97	0.92	0.94
1	Roof	0.96	**0.98**	0.97
2		**0.98**	**0.98**	**0.98**
1	Shadow	**0.95**	**0.94**	**0.95**
2		0.95	0.92	0.94
1	Tank	**0.98**	**0.98**	**0.98**
2		0.95	**0.98**	0.97
1	Tree	**0.96**	0.96	0.96
2		0.94	**0.99**	0.96

*Model No. 1 and 2 indicate the first hybrid model and the second hybrid model, respectively

Significant values are in bold

Table 3 tabulates the comparison of the precision, recall, and F1 score values of the two models, highlighting the best values in bold. Ten classes are compared, and the abilities of the two models are shown and clarified in detail. Each row displays the class name for the Building class. The second model has values with a precision of 0.95. In the class named Debris, model 1 is the best in all measures; additionally, model 2 outperforms model 1 in the class named Roof. In Shadow Class, Model 1 is the best. In Tank class, model 1 supersedes model 2 in two measures and is equal in recall with a value of 0.98. Finally, model 1 has a better precision measure with a value of 0.96, while model 2 has a better recall measure with a VAA value of 0.99. To sum up, this comparison proves that the two models are contributing to a perfect aerial image classification. Additionally, model 1 performs slightly better than model 2.

**Fig. 5:**
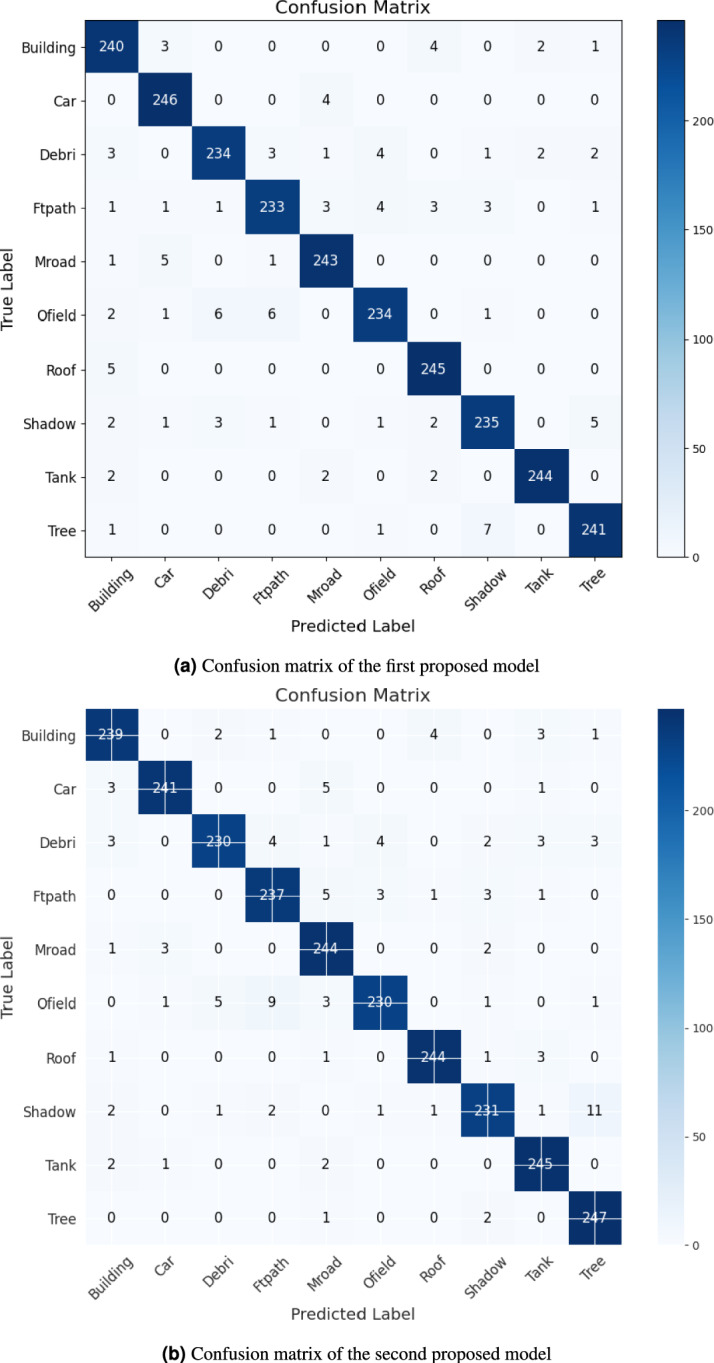
Confusion matrix of the two proposed models.

The confusion matrix is a key tool for checking how well a model performs in classification tasks. In this paper, we calculate and display two confusion matrices for the two proposed hybrid models in Fig. 5. It’s clear that the diagonal of each confusion matrix is dark blue, showing the true positive values for each of the ten classes. As mentioned, there are 250 tested images for each class. It’s obvious that the diagonal of each confusion matrix is colored with a dark blue, which indicates the true positive values for each class of the ten classes. As previously discussed, we tested 250 images for each class. For the first model, it could be noticed the best or highest value is achieved in the class named car with a value of 246, and the worst is the Ftpath class with a value of 233. In Fig. 5b, it can be noticed that, for the second model, the best value appears in class Tree with a value of 247, and the worst case is in class Debris. It’s clear that each model has an advantage over some classes, which means each of them is able to obtain or detect a meaningful feature that could be employed in a perfect way and contribute to increasing its ability to achieve the highest accuracy.

**Fig. 6:**
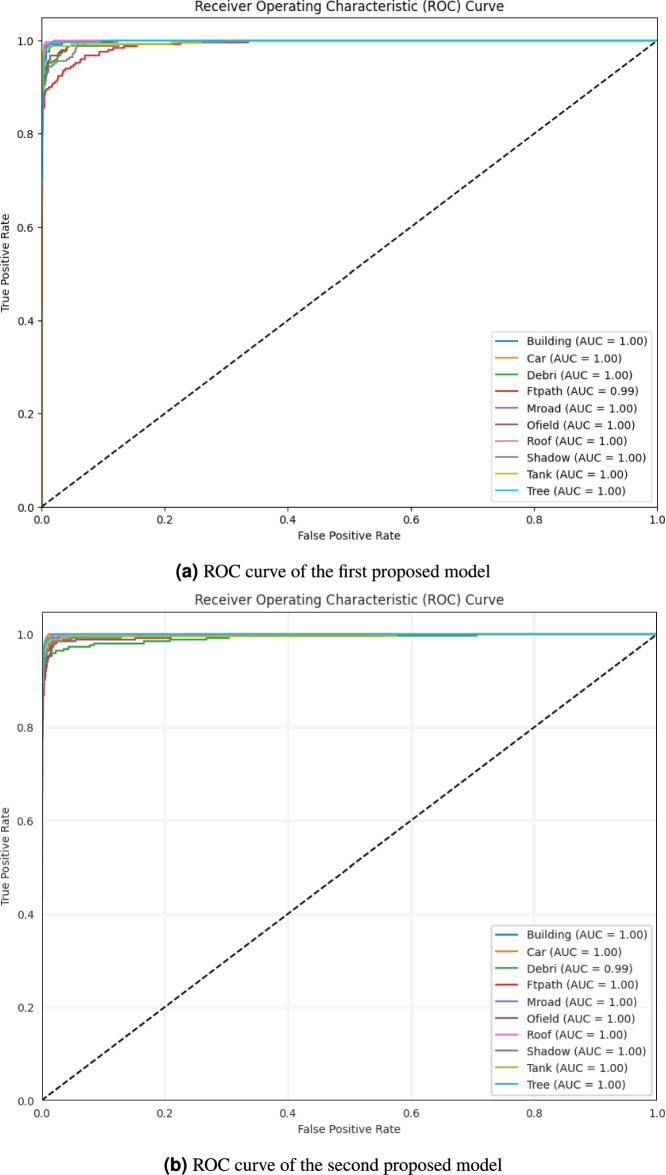
ROC curves of the two proposed models.

The receiver operating characteristics (ROC) curve is a two-dimensional graph with the true positive rate (TPR) on the y-axis and the false positive rate (FPR) on the x-axis. It’s employed to evaluate and assess various systems that have been used in different eras (e.g., machine learning systems, diagnostic systems)^26^. The optimal prediction approach would result in a point in the top left corner, or coordinate (0,1), of the ROC space, indicating 100% sensitivity (absence of false negatives) and 100% specificity (absence of false positives). The point (0,1) is referred to as a perfect categorization. To compare how well the two models work, we need to look at the ROC curves for both, which are shown in Fig. 6; the first model’s curve is in Fig. 6a and the second model’s curve is shown in Fig. 6b. There is strong evidence that each model contributes perfectly to this task; however, the first model in class Ftpath achieved an AUC of 0.99, while all other classes have an AUC equal to 1.00. The second model achieves an AUC value of 1.00 for all classes, with the exception of class Debris. This figure clearly demonstrates the superior classification performance of both models.

### Comparison with the state-of-arts methods

This section is designed for comparison with the state-of-the-art methods. The comparison in terms of the accuracy measure is shown in Table 4. All compared methods are from^12^. This table is split into three parts; the first one is comparing the basic deep learning models (e.g., AlexNet, SqueezeNet, GoogleNet, and ResNet-50) that achieved accuracy with values of 85.92, 88.52, 89.40, and 86.40, respectively. It’s clear that all values are less than 90%. While in the second part, all models based on the transformers, like ViT and Swin Transformer, have obvious results that are equal to 90% or greater than 90%. SwinSight Net was developed and achieved 93.16 %, while the YOLO based models achieved 95.33 %. Two hybrid models achieved remarkable results: 95.52% and 95.80%, respectively. It’s clear that integration between the advantages of both ResNet-50 and ViT, followed by multi-head attention or cross-attention, increases the accuracy by using the most meaningful features.

**Table 4:** Comparison with state-of-the-art methods on the SAIOD dataset.

Method	Accuracy (%)
Pretrained models	
AlexNet^12,27^	85.92
SqueezeNet^12,28^	88.52
GoogleNet^12,29^	89.40
ResNet-50^12,20^	86.40
Transformer-based models	
ViT^12,22^	90.00
Swin-transformer^12,30^	90.40
SwinSight Net (Pradhan et al.^12^)	93.16
Yolo-based models	
Pradhan et al.^13^	95.33
Proposed models	
Proposed model cross attention	**95.52**
Proposed model based MHA	**95.80**

Significant values are in bold

### Economic aspects of the hybrid models

It is worth mentioning that the proposed hybrid models efficiently classify the SAIOD dataset and cover 10 image types, contributing to sustainability. A crucial consideration, however, is the economic efficiency of the various hybrid models used for classifying different types of aerial images. which contributes significantly and effectively to smart city applications, such as smart traffic systems, road monitoring, and other challenges in smart communities. They could be integrated with the Internet of Things (IoT) or other technologies, such as satellite-based systems, to develop sustainable real-time-based applications at national levels.

## Conclusion and future work

This paper introduces two hybrid models that integrate the well-known residual network (ResNet-50), a convolutional neural network (CNN), and Vision Transformer (ViT), combined with multi-head attention or cross-attention mechanisms, to improve the aerial image classification by effectively extracting key features from diverse aerial images. The strength of these hybrid models lies in their ability to combine the advantages of both CNN and ViT and attention mechansims, addressing the challenges posed by the diverse attributes present in these ten classes of aerial images. The proposed models achieved testing accuracy values of 95.52% and 95.80% when compared to the best existing state-of-the-art methods. Future work could involve testing on different datasets, enabling real-time applications where optimization-based algorithms can be employed to fine-tune the hyperparameters and enhance the performance of the proposed model.

## Data Availability

The data used in this paper is publicly available via https://data.mendeley.com/datasets/vwznrr98b9/1.
